# Climate Change Impacts on the Future Distribution of Date Palms: A Modeling Exercise Using CLIMEX

**DOI:** 10.1371/journal.pone.0048021

**Published:** 2012-10-24

**Authors:** Farzin Shabani, Lalit Kumar, Subhashni Taylor

**Affiliations:** Ecosystem Management, School of Environmental and Rural Science, University of New England, Armidale, Australia; Plymouth University, United Kingdom

## Abstract

Climate is changing and, as a consequence, some areas that are climatically suitable for date palm (*Phoenix dactylifera* L.) cultivation at the present time will become unsuitable in the future. In contrast, some areas that are unsuitable under the current climate will become suitable in the future. Consequently, countries that are dependent on date fruit export will experience economic decline, while other countries’ economies could improve. Knowledge of the likely potential distribution of this economically important crop under current and future climate scenarios will be useful in planning better strategies to manage such issues. This study used CLIMEX to estimate potential date palm distribution under current and future climate models by using one emission scenario (A2) with two different global climate models (GCMs), CSIRO-Mk3.0 (CS) and MIROC-H (MR). The results indicate that in North Africa, many areas with a suitable climate for this species are projected to become climatically unsuitable by 2100. In North and South America, locations such as south-eastern Bolivia and northern Venezuela will become climatically more suitable. By 2070, Saudi Arabia, Iraq and western Iran are projected to have a reduction in climate suitability. The results indicate that cold and dry stresses will play an important role in date palm distribution in the future. These results can inform strategic planning by government and agricultural organizations by identifying new areas in which to cultivate this economically important crop in the future and those areas that will need greater attention due to becoming marginal regions for continued date palm cultivation.

## Introduction

Climate is one of the principal aspects defining the potential range of plants and climate change directly affects the distribution of species [1]. Much evidence exists that the climate is changing globally, and land surface temperatures are expected to increase by 4°C between the present and 2100 [2]. Moreover, worldwide seasonal rainfall patterns are changing [2]. As a consequence, a number of serious issues arise. For example, the extent of pollution and aeroallergens will change [3]. Changes in the expansion and transmission of some infectious diseases, famine, crop failure, water shortages and population displacement are some of the other issues involved with climate change. Climate change clearly threatens different areas, such as biodiversity, agricultural production, and human health. For example, it is expected that by 2030, the risk of diarrhea will increase by 10% in some specific regions due to climate change [3]. Climate change can also have an impact on agricultural production by affecting the distribution of economically important crops due to changes in their physiology [4]. The annual income from date palms in the Middle Eastern countries decreased between 1990 and 2000 [5]. A number of factors could be involved in this reduction, and climate change could be one of them because significant losses in yield of some economically important crops have been attributed to plant diseases resulting from climate change [5]. It has been reported that climate change has caused a $438 million loss in wheat, $116 million in grapes and $67 million in sugar production in Australia and North America [6].

**Figure 1:**
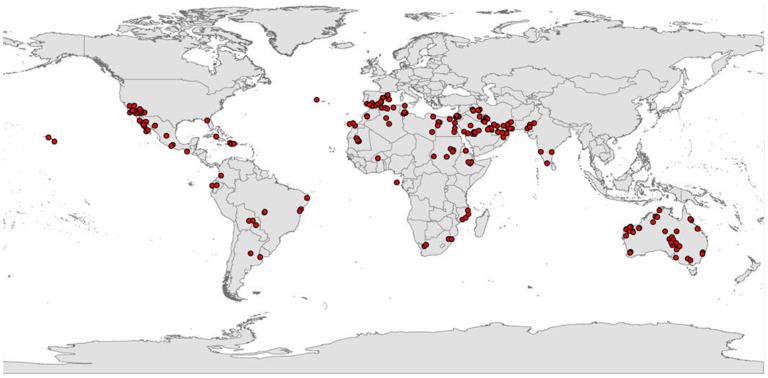
The current global distribution of *P. dactylifera*.

Date palm (*Phoenix dactylifera* L.) is a valuable plant that provides a significant source of income for both local farmers and governments in arid and semi-arid regions of the world [7]. A number of reports document the cultivation of date palms back to the 5th millennium BC. Since ancient times, the majority of date palms have continued to be grown in the hot deserts of North Africa and the Middle East, including Syria, the Persian Gulf region and north Yemen [8]. The native range of this species is from the south-eastern Azores to Pakistan, and its cultivation stems from the 4th millennium BC in Mesopotamia and Palestine [9]. The genus *Phoenix* includes up to 400 species [10]–[12] within the Arecaceae family. To mature, the fruit requires prolonged summer heat. Rain or high humidity during fruiting increases the risk of the fruit cracking and the onset of fungal diseases [13]. Long summers with high day and night temperatures, and mild, sunny, dry winters without prolonged frost are the ideal climatic conditions for this species [14].

**Table 1:** CLIMEX parameter values used for *L. dactylifera* modeling.

Parameter	Mnemonic	Values
Limiting low temperature	DV0	14°C
Lower optimal temperature	DV1	20°C
Upper optimal temperature	DV2	39°C
Limiting high temperature	DV3	46°C
Limiting low soil moisture	SM0	0.007
Lower optimal soil moisture	SM1	0.013
Upper optimal soil moisture	SM2	0.81
Limiting high soil moisture	SM3	0.9
Cold stress temperature threshold	TTCS	4°C
Cold stress temperature rate	THCS	−0.01 week^−1^
Heat stress temperature threshold	TTHS	46°C
Wet stress threshold	SMWS	0.9
Wet stress rate	HWS	0.022 week^−1^
Heat stress accumulation rate	THHS	0.9 week^−1^

Long-term management strategies to sustain economically important crops require information about the expected potential distribution and relative abundance of this plant under current and future climate scenarios. There are several distribution models that can provide information in this area, including species distribution models (SDMs), bioclimatic models and ecological niche models (ENMs). However, it has been reported that niche models only enable estimates of a species’ fundamental niche [15] while other reports show that it provides a spatial image of the realized niche [16], [17].

CLIMEX has been widely used in many different applications [18]. Taylor [19] used CLIMEX for illustrating the potential distribution of *Lantana camara L.* by 2070. Yonow [20] employed CLIMEX for mapping the distribution of the Queensland fruit fly. Sutherst [21] applied the same software for modular modeling of pests. The susceptibility of both animal and human health to parasites under future climates has also been studied using CLIMEX [22].

As a consequence of climate change, the distribution of species like date palm will change [3]. It is essential to identify which regions will benefit by having the potential opportunity of cultivating date palms in the future and which may be adversely affected. Governments and agricultural organizations can prepare for this situation in advance and thereby gain significant economic advantages which can enable them to improve their economies. Alternately, regions that could be adversely affected can become aware of the situation and transition their economies. This awareness provides an opportunity to plan for alternative sources of income. With this aim, this study made use of the CLIMEX software package in developing a global model of the climate response of *P. dactylifera* based on its native and cultivated distribution. This model was then used to illustrate date palm potential distribution using two global climate models (GCM) including CSIRO-Mk3.0 and MIROC-H. These were run with the A2 SRES (Special Report on Emissions Scenarios) emission scenarios for 2030, 2050, 2070 and 2100. The A2 SRES was chosen with the assumption that, in the future, there would be high population growth coupled with slow economic growth and extensive technological change.

**Figure 2:**
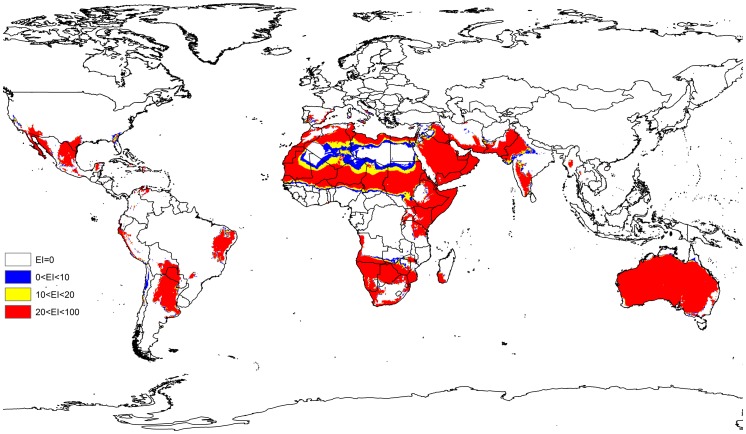
The Ecoclimatic Index for *P. Dactylifera,* modeled using CLIMEX for current climate.

## Methodology

### CLIMEX Software

CLIMEX is a modeling software package that basically operates on an eco-physiological growth model that assumes that species encounter favorable and unfavorable seasons. Growth is maximized during favorable seasons and minimized during unfavorable seasons [23]–[25]. A major criticism of CLIMEX is that it does not include biotic interactions and dispersal in the modeling process. However, other factors may be incorporated after the CLIMEX modeling has been performed using GIS and RS software [26]. The key assumption behind CLIMEX is that climate is the main determinant of the distribution of plants and poikilothermal animals [27]. CLIMEX enables the user to infer parameters that describe the species’ response to climate based on its geographic range or phenological observations [23]. The Ecoclimatic Index (EI) is a general annual index of climatic suitability based on weekly calculations of growth and stress indexes. It is scaled from 0 to 100, and theoretically, species can establish if EI >0. In CLIMEX, the annual growth index (GI_A_) describes the potential for population growth during favorable climate conditions. The GI_A_ index is determined from the temperature index (TI) and moisture index (MI) which represent the species’ temperature and moisture requirements for growth. The user can describe the probability of survival of the species during unfavorable conditions using four stresses: cold, heat, dry and wet. Therefore, based on available distribution data, this software was used to develop a model of the potential distribution of *P. dactylifera* under current and future climate scenarios.

**Figure 3:**
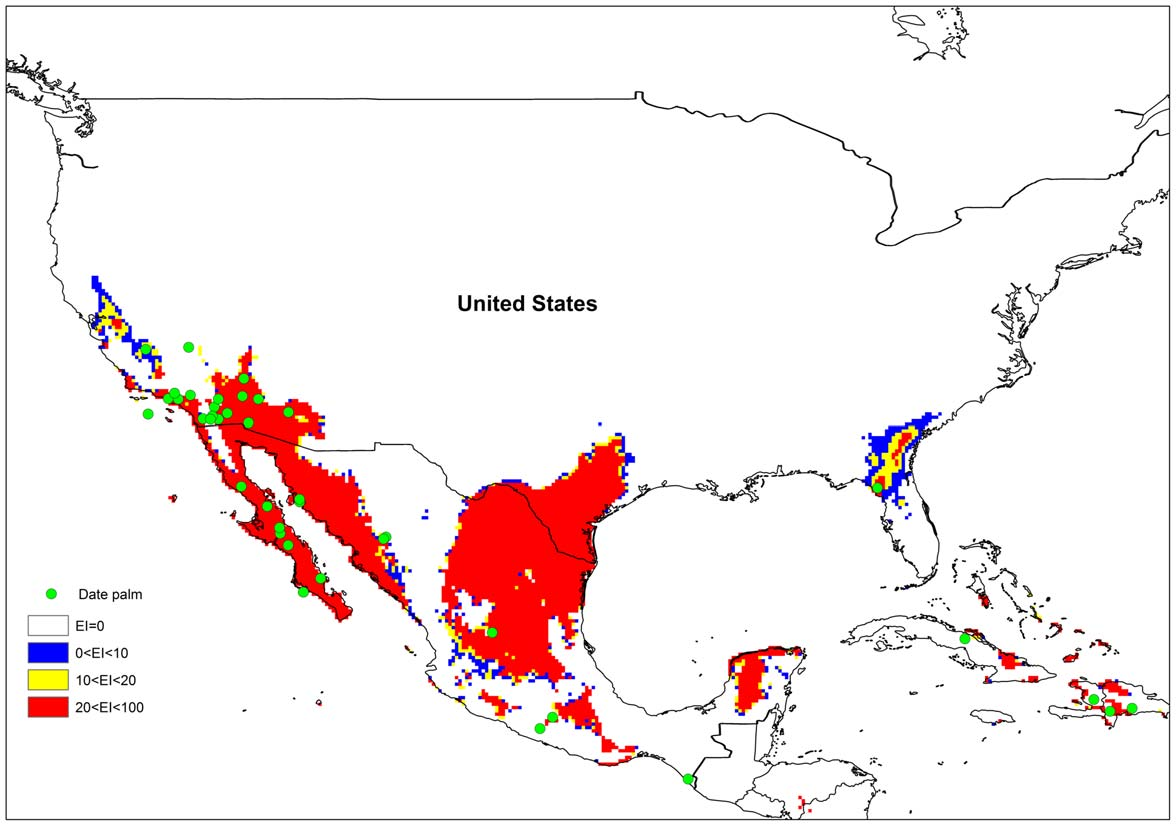
Current and potential distribution of *P. dactylifera* in validation region based on EI index.

### Distribution of Date Palms (*P. dactylifera*)

The Global Biodiversity Information Facility (GBIF) [28] was used to gather information on *P. dactylifera* distribution and this information was supplemented by other date palm literature [8], [12], [14], [28]–[41] (Figure 1). The GBIF database contained 583 records for *P. Dactylifera*; however, 342 records did not have geographic coordinates and were removed, leaving 241 records. Duplicate records were also removed. Thus, 163 records from the GBIF database and 49 records obtained from the literature review were used in parameter fitting. These 163 records were geographically representative of the known distribution of date palms as shown in Figure 1.

**Figure 4:**
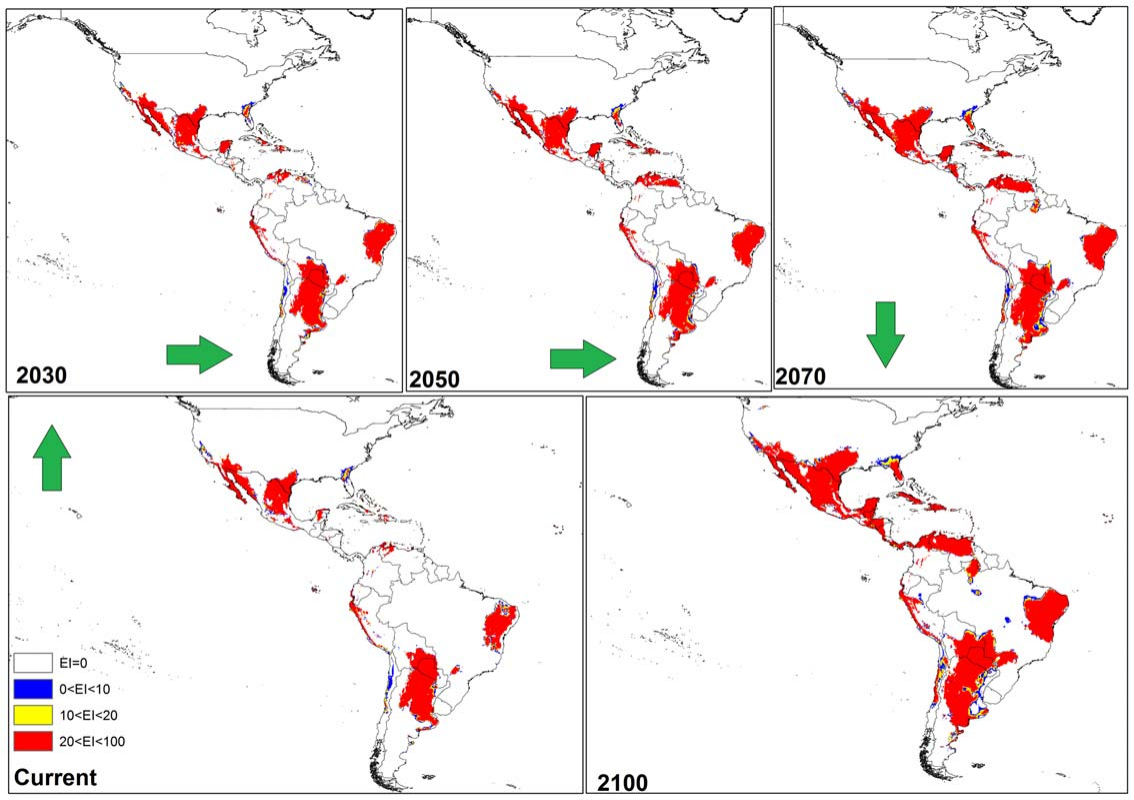
The climate (EI) for *P. dactylifera* in current time and projected using CLIMEX under the CSIRO-Mk3.0 GCM running the SRES A2 scenario and for 2030, 2050, 2070 and 2100 for the North and South America continent.

### Climate Data, Climate Models and Climate Scenarios

In this study, the CliMond 10′ gridded climate data were used for modeling [42]. Five climatic variables were utilized to represent historical climate (averaging period 1950–2000). These were average minimum monthly temperature (Tmin), average maximum monthly temperature (Tmax), average monthly precipitation (Ptotal) and relative humidity at 09∶00 h (RH09∶00) and 15∶00 h (RH15∶00). These variables were also used to typify potential future climate in 2030, 2050, 2070 and 2100. The potential distribution of date palms under future climate was modeled using two Global Climate Models (GCMs), CSIRO-Mk3.0 [42] and MIROC-H (Center for Climate Research, Japan), with the A2 SRES scenario [42]–[44]. These two GCMs were part of the CliMond dataset and were selected from 23 GCMs based on the following criteria:

All required variables, including temperature, precipitation, sea level pressure and humidity for CLIMEX were available
Small horizontal grid spacing in both GCMs
Better representation of observed climate at local scales, compared to the other GCMs [45].

In the remainder of this paper, MR and CS are used as the abbreviation of MIROC-H and CSIRO-Mk3.0, respectively.

**Figure 5:**
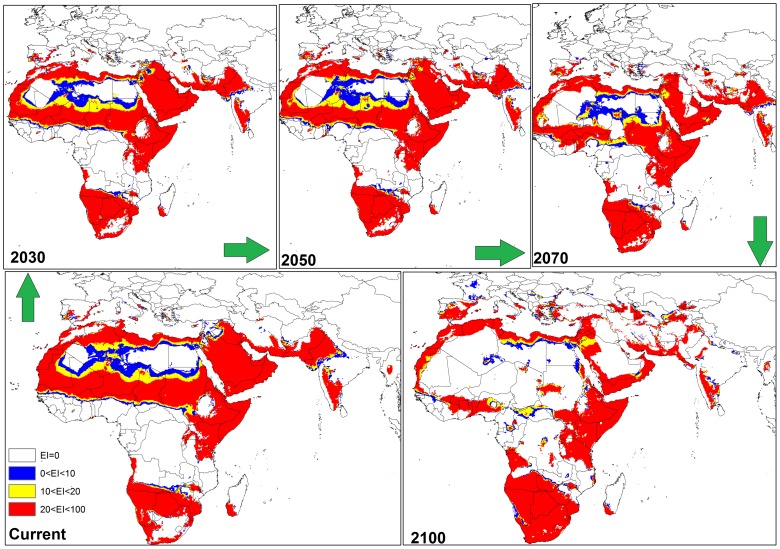
The climate (EI) for *P. dactylifera* in current time and projected using CLIMEX under the CSIRO-Mk3.0 GCM running the SRES A2 scenario and for 2030, 2050, 2070 and 2100 for the north and south of Africa and the Middle East.

The MR model predicts that temperature will increase by approximately 4.31°C, while the CS model predicts a rise of 2.11°C by 2100. There are also differences in rainfall patterns for CS and MR models. For example, the CS model predicts a 14% decrease in future mean annual rainfall, whereas the MR model predicts a 1% decrease [46], [47].

The A2 scenario was selected to characterize one of the possible climate scenarios during 2030, 2050, 2070 and 2100. The A2 scenario covers different factors including demographic, economic and technological forces driving GHG emissions; this scenario assumes neither very high nor low global GHG emissions compared to the other scenarios, such as A1F1, A1B, B2, A1T, B1 by 2100 [47].

No scenarios from the B family of SRES scenarios were included in this paper, mainly because of the observation that some parameters such as global temperature and sea level rise are presently increasing at a much greater rate than predicted by the hottest SRES scenarios [48].

**Figure 6:**
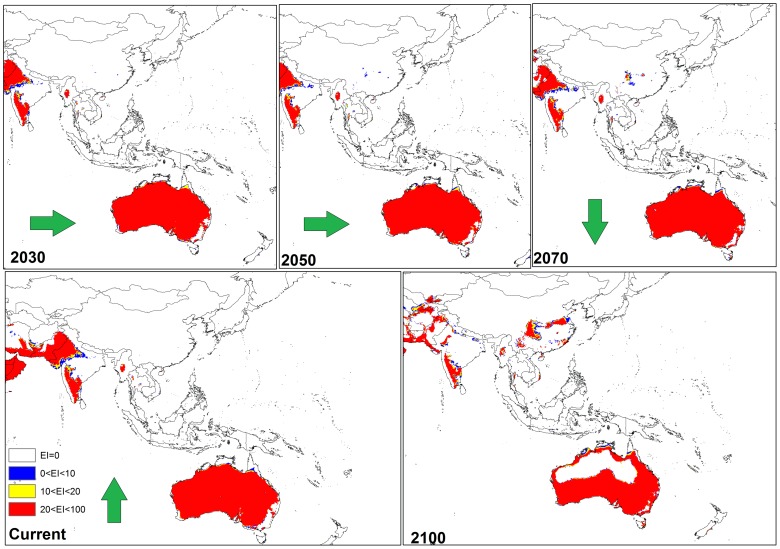
The climate (EI) for *P. dactylifera* in current time and projected using CLIMEX under the CSIRO-Mk3.0 GCM running the SRES A2 scenario and for 2030, 2050, 2070 and 2100 for Australia, and southern Asia.

### Fitting CLIMEX Parameters

Using both native habitat range and agricultural distribution data in parameter fitting is highly recommended because it produces a model that approximates the potential distribution of the taxa being modeled [49]. This is because the limitations imposed by biotic influences in the species’ native range may be absent in non-native locations, thus allowing it to expand its range beyond its realized Hutchinsonian niche [49], [50]. In this study, parameters were fitted using the native range and the global agricultural distribution of date palms. However, the distribution data of *P. dactylifera* from North America, Mexico, and the Caribbean were not used in parameter fitting as this was set aside for model validation. The parameters were iteratively adjusted depending on satisfactory agreement between the potential and known worldwide distribution of *P. dactylifera*. The parameters were subsequently verified to ensure that they were biologically reasonable. Model validation was conducted using North American, Mexican, and Caribbean distribution data. It should be highlighted that the wet stress threshold parameter does not have a unit, while the stress accumulation rate uses the week^−1^ unit. The heat and cold stress thresholds use degrees Celsius (°C) unit.

**Figure 7:**
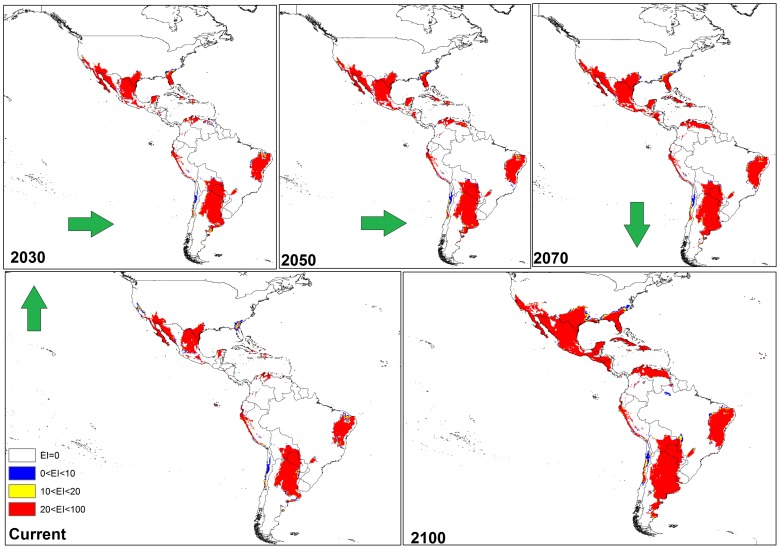
The climate (EI) for *P. dactylifera* in current time and projected using CLIMEX under the MIROC-H GCM GCM running the SRES A2 scenario and for 2030, 2050, 2070 and 2100 for the North and South America continent.

### Cold Stress

The cold stress temperature threshold (TTCS) mechanism was used to describe the species’ response to frost. Generally, the minimum winter temperature that can be tolerated by *P. dactylifera* is 10°C [11]. However, date palms have been recorded in locations as low as 4°C [28]. Therefore, intolerance to frost was incorporated by accumulating stress when the average monthly minimum temperature fell below 4°C, with the frost stress accumulation rate (THCS) set at –0.01 week^−1^. This cold-stress mechanism allowed the species to survive in Spain (39° 635′ N and 2° 523′ W) [28]. Additionally, this value provided an appropriate fit to the observed distribution in South America, South Africa and Asia.

**Figure 8:**
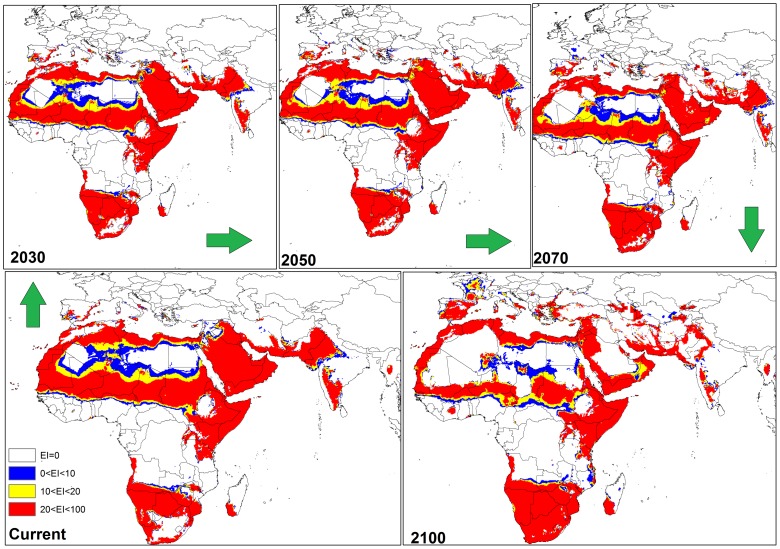
The climate (EI) for *P. dactylifera* in current time and projected using CLIMEX under the MIROC-H GCM running the SRES A2 scenario and for 2030, 2050, 2070 and 2100 for the northern and southern Africa and the Middle East.

### Heat Stress

The heat stress parameter (TTHS) was set at 46°C because it was reported that *P. dactylifera* is able to persist up to this temperature in eastern Pakistan [28]. The heat stress accumulation rate (THHS) was set at 0.9 week^−1^, which allowed *P. dactylifera* to persist along eastern Pakistan [37], [38], [42] and southern Iran [8], [28], [31].

### Dry Stress

The term ‘drought’ refers to a period of time without significant rainfall [14]. Water stress occurs as a consequence of water loss through transpiration or evaporation during a period of time when there is a lack of available water in the soil [14]. Different degrees of water stress can be seen in a plant. When water loss is prolonged, a significant disruption in the metabolism of the plant occurs [14]. However, the date palm has developed a number of strategies to prevent dry stress. These include maintaining a high level of hydration, the ability to function while dehydrated, increasing the amount of water absorption (i.e., keeping a high level of osmotic pressure) by using abscisic acid, and by the development of an extensive root system [14]. Dry stress was not used in this study for the above reasons.

**Figure 9:**
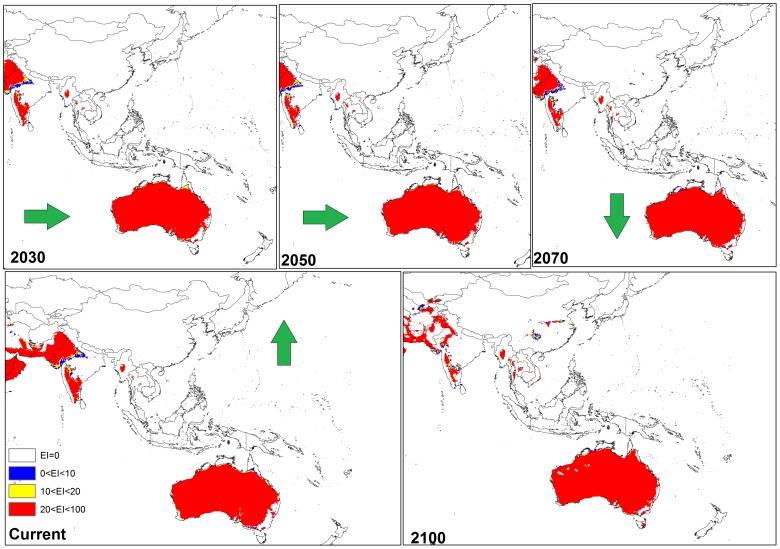
The climate (EI) for *P. dactylifera* in current time and projected using CLIMEX under the MIROC-H GCM running the SRES A2 scenario and for 2030, 2050, 2070 and 2100 for Australia and China.

### Wet Stress

August to October are the critical months when rain damage can inflict serious economic damage to the date crop [11]. A recent study observed that a total of 78.74 mm of rainfall during an 8-day period caused a greater than 50% loss in date palm yields while 86.36 mm of rainfall in 10 days led to 15% losses in date palm farms in some countries [11]. Date palms are known to suffer wet stress easily. The wet stress threshold (SMWS) was set to 0.9 and the accumulation rate (HWS) set at 0.022 week^−1^ to allow the species to grow well in arid and semi-arid regions such as Algeria, Morocco, and southern Iran.

**Figure 10:**
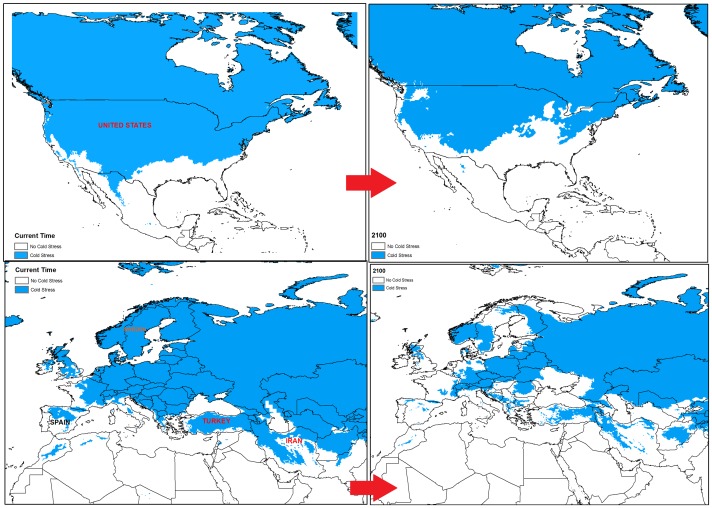
Comparison of the location of cold stress in some selected regions for date palm growth between current time and 2100. These areas were selected on the basis of large changes in cold stress in the future.

**Figure 11:**
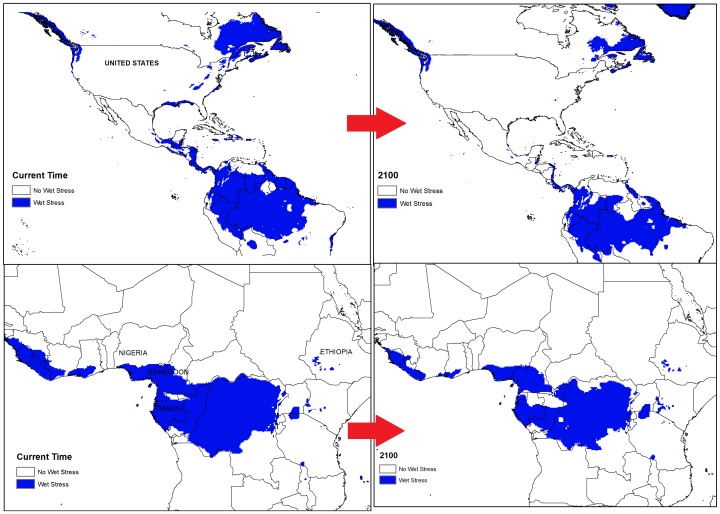
Comparison of the location of wet stress in some selected regions for date palm growth between current time and 2100. These areas were selected on the basis of large changes in wet stress in the future.

### Temperature Index

*Phoenix dactylifera* has been cultivated in areas with a mean annual surface temperature greater than 16°C, such as southern Iran [8], [28], [29], south-eastern Iraq, eastern Pakistan [11], [38], and northern and central Algeria [11], [39]. Western Pakistan’s climatic parameters are comparable to other places suitable for date palm cultivation with the exception of its annual surface temperature, which is 13°C. Thus, the limiting low temperature (DV0) should be between 13°C and 16°C. Fourteen degrees Celsius was selected due to providing the best fit to the observed distribution of date palms in North Africa and Asia. Summer temperatures in locations which are highly climatically suitable for this species rarely exceed 46°C, thus the limiting high temperature DV3 was set at 46°C [13]. The lower (DV1) and upper (DV2) optimal temperatures were set at 20°C and 39°C, respectively, because temperatures between 20°C and 39°C are cited as favorable temperatures for date palm, depending on the varieties [14]. These numbers also provided the best fit to the observed distribution in South America, Asia, South Africa and Australia [11].

### Moisture Index

In terms of soil moisture, the lower moisture threshold (SM0) was set at 0.007, to represent the permanent wilting point [27]. Furthermore, this number provided a good fit to the observed distribution of date palms in South America, Asia and the Middle East. The lower (SM1) and upper (SM2) optimum moisture thresholds were set at 0.013 and 0.81, respectively, to improve species growth in Egypt, Saudi Arabia, Iran, India, and some countries in Africa [11]. The upper soil moisture threshold (SM3) was set at 0.9 because this species and its fruit can be negatively affected by high soil moisture [11]. Additionally, this value provided an appropriate fit to the observed distribution. All CLIMEX parameters are summarized in Table 1.

## Results

### Current Climate

The present distribution of native and cultivated *P. dactylifera* is illustrated in Figure 1. A comparison between the modeled global climate appropriateness (Figure 2) with the recognized distribution of this species showed that there was a good match between the Ecoclimatic Index resulting from the CLIMEX model and the current distribution of *P. dactylifera*. The modeled results indicated that the western areas of the United States, western Mexico, southeast Spain, Morocco, Portugal, central Sudan, Egypt, eastern Mozambique, central and western United Arab Emirates, southern Iran, eastern Pakistan and large parts of Australia have suitable climatic conditions for *P. dactylifera*.

Although large parts of central southern Africa were modeled to have suitable climatic conditions for *P. dactylifera* in its current known distribution, limited data were available from these regions. This could be due to a shortage of reports from these areas. Biotic factors such as competition or lack of dispersal opportunities could preclude this species from occurring in these areas. There is also a possibility that date palm has not been grown as an economically important crop in those regions.

The current and potential distribution of *P. dactylifera* in North America, Mexico, and the Caribbean was used for model validation as shown in Figure 3. These regions were not used for model fitting. In Mexico and North America, the model projects much of the southern and south-western coast to be climatically suitable. There was a reasonably good fit between the model predictions and the actual recorded distribution data.

### Future Climate

The results of the two global climate change models (GCMs) including CSIRO-Mk3.0 (CS) and MIROC-H (MR) with the A2 emission scenarios for the potential distribution of *P. dactylifera* for 2030, 2050, 2070 and 2100 are illustrated in Figures 4, 5, 6, 7, 8 and 9. For ease of discussion, the global distribution is subdivided into three regions: North and South America, Africa and Middle East, and Australia and South Asia.

#### a) Results from CS model

In North and South America (Figure 4), the CS GCM projected much of the south-western coast of Mexico and North America, eastern Brazil, south-eastern Bolivia, northern Venezuela, Cuba, northern Colombia, and Paraguay to be more climatically suitable for *P. dactylifera* by 2030; this expansion steadily increased by 2050, 2070 and 2100. Interestingly, from northern Venezuela to the central regions, the climate was predicted to be highly suitable for date palms by 2100.

In southern Africa (Figure 5), the CS model predicted an expansion of the range of *P. dactylifera* further inland from now to 2100. In North Africa, particularly in central and southern Algeria, Mauritania, Mali, Niger, all of the Sudan excluding the western side, and southern Tunisia were projected to become progressively less suitable (EI = 0 or EI<1–10) by 2070 and totally unsuitable by 2100 (Figure 5).

The CS GCM for the Middle East indicated that by 2030, Saudi Arabia, Iraq and western Iran would remain climatically suitable (Figure 5). However, by 2050 a reduction in climate suitability for *P. dactylifera* was predicted for all three countries; this trend was particularly accentuated in Saudi Arabia and Iraq by 2100 (Figure 5). In Asia, especially in northern India, eastern Pakistan and southern Afghanistan (Figure 5), and in north-western Australia (Figure 6), there was a considerable reduction in climate suitability for date palms between 2050 and 2100.

#### b) Results from MR model

From the MR GCM, it can be seen that in the Americas, much of the south-western coast of Mexico, North America, eastern Brazil, south-eastern Bolivia, northern Venezuela, Cuba, northern Colombia, and Paraguay are projected to become climatically suitable for date palms between 2030 and 2100 (Figure 7). Moreover, the MR GCM predicted that more areas around Florida may become suitable for this species’ growth by 2100 (Figure 7). The MR GCM projected that by 2100, western Argentina would be more climatically suitable than it is currently.

The MR GCM predicted that almost all of the southern regions on the African continent may become suitable for *P. dactylifera* in the future (Figure 8). In contrast, some countries in North Africa such as Algeria, Mali, Niger, Mauritania and Sudan are projected to become progressively less suitable, with date production becoming completely unviable by 2100 (Figure 8). However, this model projected that some countries such as Namibia, Botswana and parts of southern Zambia are likely to become highly suitable, particularly from 2070 onwards (Figure 8).

The MR GCM for the Middle East projected that Saudi Arabia, Iraq and western Iran may remain climatically suitable for date palms until 2050 (Figure 8). However, the model projected that by 2070 the climate of Saudi Arabia, Iraq and western Iran would be significantly less suitable and that by 2100, the climate in large parts of Saudi Arabia and Iraq would be unsuitable for date palm cultivation. Moreover, a considerable reduction in suitability of climate for date palms was found from 2050 to 2100 in Asia, particularly in northern India, eastern Pakistan and southern Afghanistan (Figure 8).

The results indicated that there were some differences in the projection of date palm distribution between the CSIRO-Mk3.0 and MIROC-H GCMs. These differing results were due to the varying predictions of future climate by the two GCMs.

Based on the two models, cold and wet stresses appear to be the major factors restricting date palm distribution. For example, cold stress is currently the main limiting factor in Canada, most parts of the United States (excluding Florida and California), Peru, Chile, and Ecuador, south-eastern Australia and most areas of China (Figures 7, 8 and 9). The same results were found for central to western Mali as a consequence of heat stress, which imposes a significant limitation for date palm establishment. Additionally, due to wet stress, *P. dactylifera* cannot be successfully grown in areas of eastern South America, such as central Guatemala, Colombia, Uruguay, and southern Chile, nor in parts of southern Africa including Angola, Zambia, Zimbabwe, and northern Madagascar. Wet stress also causes Germany, Poland, Ireland, northern Portugal, Azerbaijan, Georgia, southern India, Thailand, Burma, Bhutan, eastern Nepal, Spain and southern eastern Australia to be unsuitable for the establishment of this species. Thus, cold and wet stresses impose significant limitations for expanding the global distribution of date palm in 2030 and beyond. The current and projected distribution of cold and wet stresses can be seen for selected regions in Figures 10 and 11, respectively. In the United States, the cold and wet stresses shift northward, meaning there may be larger areas available that are not affected by the aforementioned stresses and therefore more are conducive to date palm cultivation. Our modeling showed that cold and wet stresses will no longer be the limiting factors in large parts of the United States.

## Discussion

Suitable climatic areas for *P. dactylifera* under present and future climate scenarios using CLIMEX were modeled in this study. The differences in the outcomes from the two GCMs emphasize the uncertainties associated with the state of climate modeling associated with greenhouse emission patterns [14]. It is clear that different models may produce different results. It should also be highlighted that suitability projections are only potential distributions based on climatic factors and not predicted future distributions [14]. Thus, it is highly recommended that any projection of future suitable areas based on CLIMEX should also incorporate non-climatic factors such as land-use type, soil type, soil drainage and soil-nutrients [11].

Here, our model provided a good fit to the present global distribution records of date palm on the southern coast of Mexico and south-western North America, regions that were used to validate the model.

In this study, both CS and MR GCMs projected that in the Americas, some regions including the south-western coast of Mexico and North America, eastern Brazil, south-eastern Bolivia, northern Venezuela, Cuba, northern Colombia and Paraguay will become more climatically suitable towards 2100. However, the MR GCM projected Florida becoming more climatically suitable than the CS GCM between 2030 and 2100 due the projection of a greater increase in temperature and smaller decrease in the amount of rainfall in the MR GCM [46], [47]. Thus, date palms would not suffer any wet or cold stress in Florida. A comparison between these two models also indicated that, based on the MR GCM, more regions in western Argentina may be suitable for date palm growth compared to the CS projection (Figures 4 and 7). A comparison between the results of CS and MR GCM for southern Africa indicated that *P. dactylifera* ranges appeared to shift further inland in the future. However, the CS GCM projected that most regions in Angola may be climatically suitable by 2100, but, based on MR GCM, this suitability may be limited to the southern and coastal regions due to an increase in the wet stress in northern and eastern Angola (Figures 5 and 8).

There were some divergent results in the projection of suitable areas for date palms in North Africa and Middle Eastern countries between the CS and MR GCMs. For example, both models projected that northern Algeria, Morocco, Western Sahara, Tunisia, northern Egypt, Somalia and Kenya may become climatically suitable for *P. dactylifera*. Furthermore, both models projected that southern Algeria, eastern Mauritania, northern Mali and western Chad may become unsuitable for this species. In contrast, the CS model projected that by 2100, Mali, Niger, Chad and most parts of Sudan may not be suitable for date palm, while the MR model projected that southern Mali and Niger, eastern Chad and western Sudan may remain climatically suitable for date palms by 2100 (Figures 5 and 8).

There were some agreements and disagreements in projection of suitable areas for date palm growth in North Africa and Middle Eastern countries between CS and MR GCMs. For example, both models projected that northern Algeria, Morocco, western Sahara, Tunisia, northern Egypt, Somalia and Kenya may become climatically suitable for *P. dactylifera* growth. Furthermore, both models projected that southern Algeria, eastern Mauritania, northern Mali and western Chad may be unsuitable for this species. On the other hand, the CS model projected that by 2100, Mali, Niger, Chad and most parts of Sudan may become unsuitable for date palm growth since the MR model projected that southern Mali and Niger, eastern Chad and western Sudan may remain climatically suitable for date palms growth by 2100 (Figures 5 and 8).

From the results (Figures 10 and 11), it is evident that currently unsuitable areas such as the western United States, southern Mexico, northern and southern Africa, may become suitable climatically by 2100 through decreasing cold and wet stresses. Iran, Turkey, and Spain are some examples where cold stress may decrease by 2100 (Figure 10). Figure 11 illustrates that wet stress in northern Gabon and eastern Quebec may decrease over the next few decades.

The results of the climate change modeling provide an indication of the possible change in the potential future distribution of *P. dactylifera.* As the climate changes, some areas where *P. dactylifera* currently occurs may become climatically unsuitable, and as a consequence, the economies in those areas may decline. For example, it was reported that Algeria and Saudi Arabia earned 3621 and 1378 U.S. dollars/tonne, respectively, in 1995 from exporting dates. The large disparity in price was due to their strategies in targeting different countries and the differences in date quality [11]. However, this study indicates that large areas of Algeria and Saudi Arabia may become climatically unsuitable and may not be able to cultivate this profitable crop to the same extent in the future. The results are in line with current observations of a decline in date palm production in Middle Eastern countries from 1990 to 2000 [4], [5].

Consequently, the results of this paper provide some advance awareness for countries which rely on income from exporting dates. Furthermore, by making some strategic plans, many economic disadvantages can be prevented. This information is particularly important for some countries in northwestern Africa and the Middle East.

Conversely, the results indicate that some areas which are climatically unsuitable at present may become suitable for date palm cultivation in the future. These outcomes may well be useful in making informed choices about the location of date palm farms and associated industries in advance. Benin, Ghana, Cameroon, Nigeria, Venezuela and China may have the opportunity to cultivate date palms and export its produce in the future. Under future climate, *P. dactylifera* may be able to be cultivated in areas that are currently too cold or wet; this can be seen in the improved climatic suitability for Florida, Mexico, northern Venezuela, and eastern Brazil in the Americas; South Sudan and Guinea in Africa; and Spain and France in Europe. These countries should be prepared to make use of these opportunities since, climate-wise, these areas may become highly suitable for this plant. Specifically, these maps could be used by agricultural organizations in various countries to make strategic, long-term plans. This may include research into alternative crops in areas where climate will become unfavorable for date palms.

In interpreting these results, the following should be considered:

- The modeling was performed based only on climate; it does not take into consideration other factors such as land uses, soil types, biotic interactions, diseases and competition.
- This research was based on currently available broad-scale climate data; therefore, it only shows broad-scale shifts.
- It is indicative because a certain level of uncertainty is associated with future levels of greenhouse gas emissions.

In conclusion, this research has demonstrated broad-scale shifts in areas conducive to date palm cultivation and how different areas of the world may be affected due to climate change based on broad regional-scale changes over the next hundred years using coarse scale climate data. Some regions were projected to be climatically unsuitable as a consequence of only one stress for date palm growth, such as wet stress in northern Angola. However, some regions were projected to be unsuitable as a consequence of a combination of multiple stresses; for example, the combination of wet and cold stresses imposed negative effects on date palms growth in the northern United States, meaning that the effects of stresses differ regionally. Such modeling is useful in planning future strategies and minimizing economic impacts in areas that may be adversely impacted, while preparing to take advantage of new opportunities in regions that may be positively impacted.
